# Efficient Coalescent Simulation and Genealogical Analysis for Large Sample Sizes

**DOI:** 10.1371/journal.pcbi.1004842

**Published:** 2016-05-04

**Authors:** Jerome Kelleher, Alison M Etheridge, Gilean McVean

**Affiliations:** 1 Wellcome Trust Centre for Human Genetics, University of Oxford, Oxford, United Kingdom; 2 Department of Statistics, University of Oxford, Oxford, United Kingdom; 3 Li Ka Shing Centre for Health Information and Discovery, University of Oxford, Oxford, United Kingdom; UC Berkeley, UNITED STATES

## Abstract

A central challenge in the analysis of genetic variation is to provide realistic genome simulation across millions of samples. Present day coalescent simulations do not scale well, or use approximations that fail to capture important long-range linkage properties. Analysing the results of simulations also presents a substantial challenge, as current methods to store genealogies consume a great deal of space, are slow to parse and do not take advantage of shared structure in correlated trees. We solve these problems by introducing sparse trees and coalescence records as the key units of genealogical analysis. Using these tools, exact simulation of the coalescent with recombination for chromosome-sized regions over hundreds of thousands of samples is possible, and substantially faster than present-day approximate methods. We can also analyse the results orders of magnitude more quickly than with existing methods.

## Author Summary

Our understanding of the distribution of genetic variation in natural populations has been driven by mathematical models of the underlying biological and demographic processes. A key strength of such coalescent models is that they enable efficient simulation of data we might see under a variety of evolutionary scenarios. However, current methods are not well suited to simulating genome-scale data sets on hundreds of thousands of samples, which is essential if we are to understand the data generated by population-scale sequencing projects. Similarly, processing the results of large simulations also presents researchers with a major challenge, as it can take many days just to read the data files. In this paper we solve these problems by introducing a new way to represent information about the ancestral process. This new representation leads to huge gains in simulation speed and storage efficiency so that large simulations complete in minutes and the output files can be processed in seconds.

## Introduction

The coalescent process [1, 2] underlies much of modern population genetics and is fundamental to our understanding of molecular evolution. The coalescent describes the ancestry of a sample of *n* genes in the absence of recombination, selection, population structure and other complicating factors. The model has proved to be highly extensible, and these and many other complexities required to model real populations have successfully been incorporated [3]. Simulation has played a key role in coalescent theory since its beginnings [2], partly due to the ease with which it can be simulated: for a sample of *n* genes, we require only *O*(*n*) time and space to simulate a genealogy [4].

Soon after the single locus coalescent was derived, Hudson defined an algorithm to simulate the coalescent with recombination [5]. However, after some early successes in characterising this process [6, 7] little progress was made because of the complex distribution of blocks of ancestral material among ancestors. Some years after Hudson’s pioneering work, the study of recombination in the coalescent was recast in the framework of the Ancestral Recombination Graph [8, 9]. In the ARG, nodes are events (either recombination or common ancestor) and the edges are ancestral chromosomes. A recombination event results in a single ancestral chromosome splitting into two chromosomes, and a common ancestor event results in two chromosomes merging into a common ancestor. Analytically, the ARG is a considerable simplification of Hudson’s earlier work as it models all recombination events that occurred in the history of a sample and not just those that can potentially affect the genealogies. Many important results have been derived using this framework, one of which is particularly significant for our purposes here. Ethier and Griffiths [10] proved that the expected number of recombination events back to the Grand MRCA of a sample of *n* individuals grows like *e*^*ρ*^ as *ρ* → ∞, where *ρ* is the population scaled recombination rate. In this paper we consider a diploid model in which we have a sequence of *m* discrete sites that are indexed from zero. Recombination occurs between adjacent sites at rate *r* per generation, and therefore *ρ* = 4*N*_*e*_ *r*(*m* − 1). The Ethier and Griffiths result implies that the time required to simulate an ARG grows exponentially with the sequence length, and we can only ever hope to simulate ARGs for the shortest of sequences.

This result, coupled with the observed poor scaling of coalescent simulators such as the seminal ms program [11] seems to imply that simulating the coalescent with recombination over chromosome scales is hopeless, and researchers have therefore sought alternatives. The sequentially Markov coalescent (SMC) approximation [12, 13] underlies the majority of present day genome scale simulation [14–16] and inference methods [17–19]. The SMC simplifies the process of simulating genealogies by assuming that each marginal tree depends only on its immediate predecessor as we move from left-to-right across the sequence. As a consequence, the time required to simulate genealogies scales linearly with increasing sequence length. In practice, SMC based simulators such as MaCS [14] and scrm [16] are many times faster than ms.

The SMC has disadvantages, however. Firstly, the SMC discards all long range linkage information and therefore can be a poor approximation when modelling features such as the length of admixture blocks [20]. Improving the accuracy of the SMC can also be difficult. For example, MaCS has a parameter to increase the number of previous trees on which a marginal tree can depend. Counter-intuitively, increasing this parameter beyond a certain limit results in a *worse* approximation to the coalescent with recombination [16]. (The scrm simulator provides a similar parameter that does not exhibit this unfortunate behaviour, however.) Incorporating complexities such as population structure [21], intra-codon recombination [22] and inversions [23] is non-trivial and can be substantially more complex than the corresponding modification to the exact coalescent model. Also, while SMC based methods scale well in terms of increasing sequence length, currently available simulators do not scale well in terms of sample size.

We solve these problems by introducing sparse trees and coalescence records as the fundamental units of genealogical analysis. By creating a concrete formalisation of the genealogies generated by the coalescent process in terms of an integer vector, we greatly increase the efficiency of simulating the exact coalescent with recombination. In the section **Efficient coalescent simulation**, we discuss how Hudson’s classical simulation algorithm can be defined in terms of these sparse trees, and why this leads to substantial gains in terms of the simulation speed and memory usage. We show that our implementation of the exact coalescent, msprime, is competitive with approximate simulators for small sample sizes, and is faster than all other simulators for large sample sizes. This is possible because Hudson’s algorithm does not traverse the entire ARG, but rather a small subset of it. The ARG contains a large number of nodes that do not affect the genealogies of the sample [24], and Hudson’s algorithm saves time by not visiting these nodes. This subset of the ARG (sometimes known as the ‘little’ ARG) has not been well characterised, which makes analysis of Hudson’s algorithm difficult. However, we show some numerical results indicating that the number of nodes in the little ARG may be a quadratic function of the scaled recombination rate *ρ* rather than an exponential.

Generating simulated data is of little use if the results cannot be processed in an efficient and convenient manner. Currently available methods for storing and processing genealogies perform very poorly on trees with hundreds of thousands of nodes. In the section **Efficient genealogical analysis**, we show how the encoding of the correlated trees output by our simulations leads to an extremely compact method of storing these genealogies. For large simulations, the representation can be thousands of times smaller than the most compact tree serialisation format currently available. Our encoding also leads to very efficient tree processing algorithms; for example, sequential access to trees is several orders of magnitude faster than existing methods.

The advantages of faster and more accurate simulation over huge sample sizes, and the ability to quickly process very large result sets may enable applications that were not previously feasible. In the **Results and Discussion** we conclude by considering some of these applications and other uses of our novel encoding of genealogies. The methods developed in this paper allow us to simulate the coalescent for very large sample sizes, where the underlying assumptions of the model may be violated [25–27]. Addressing these issues is beyond the scope of this work, but we note that the majority of our results can be applied to simulations of any retrospective population model.

## Methods

### Efficient coalescent simulation

In this section we define our encoding of coalescent genealogies, and show how this leads to very efficient simulations. There are many different simulation packages, and so we begin with a brief review of the state-of-the-art before defining our encoding and analysing the resulting algorithm in the following subsections.

Two basic approaches exist to simulate the coalescent with recombination. The first approach was defined by Hudson [5], and works by applying the effects of recombination and common ancestor events to the ancestors of the sample as we go backwards in time. Events occur at a rate that depends only on the state of the extant ancestors, and so we can generate the waiting times to these events efficiently without considering the intervening generations. This contrasts with time-reversed generation-by-generation methods [28–31] which are more flexible but also considerably less efficient. The first simulation program published based on Hudson’s algorithm was ms [11]. After this, many programs were published to simulate various evolutionary complexities not handled by ms, such as selection [32–35], recombination hotspots [36], codon models [37], intra-codon recombination [22] and models of species with a skewed offspring distribution [38]. Others developed user interfaces to facilitate easier analysis [39, 40].

The second fundamental method of simulating the coalescent with recombination is due to Wiuf and Hein [24]. In Wiuf and Hein’s algorithm we begin by generating a coalescent tree for the left-most locus and then move across the sequence, updating the genealogy to account for recombination events. This process is considerably more complex than Hudson’s algorithm because the relationship between trees as we move across the genome is non-Markovian: each tree depends on all previously generated trees. Because of this complexity, exact simulators based on Wiuf and Hein’s algorithm are significantly less efficient than ms [16, 41]. However, Wiuf and Hein’s algorithm has provided the basis for the SMC approximation [12, 13], and programs based on this approach [14–16] can simulate long sequences far more efficiently than exact methods such as ms. Very roughly, we can think of Wiuf and Hein’s algorithm performing a depth-first traversal of the ARG, and Hudson’s algorithm a breadth-first traversal. Neither explore the full ARG, but instead traverse the subset required to contruct all marginal genealogies.

Recently, Hudson’s algorithm has been utilised in cosi2 [35], which takes a novel approach to simulating sequences under the coalescent. The majority of simulators first generate genealogies and then throw down mutations in a separate process. In cosi2 these two processes are merged, so that mutations are generated during traversal of the ARG. Instead of associating a partial genealogy with each ancestral segment, cosi2 maps ancestral segments directly to the set of sampled individuals at the leaves of this tree. When a coalescence between two overlapping segments occurs, we then have sufficient information to generate mutations and map them to the affected samples. This strategy, coupled with the use of sophisticated data structures, makes cosi2 many times faster than competing simulators such as msms [34]. The disadvantage of combining the mutation process with ARG traversal, however, is that the underlying genealogies are not available, and cosi2 cannot directly output coalescent trees.

Many reviews are available to compare the various coalescent simulators in terms of their features [42–47]. Little information is available, however, about their relative efficiencies. Hudson’s ms is widely regarded as the most efficient implementation of the exact coalescent and is the benchmark against which other programs are measured [13–16, 41, 47]. However, for larger sample sizes and long sequence lengths, msms is much faster than ms. Also, for these larger sequence lengths and sample sizes, ms is unreliable and crashes [15, 47]. Thus, msms is a much more suitable baseline against which to judge performance. The scrm simulator is the most efficient SMC based method currently available [16].

#### Hudson’s algorithm with sparse trees

**Fig 1:**
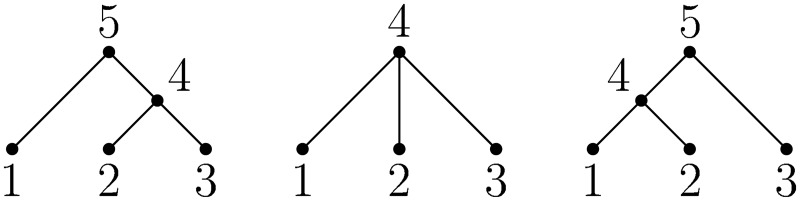
Example oriented trees. From left-to-right, these trees are defined by the sequences 〈5, 4, 4, 5, 0〉, 〈4, 4, 4, 0〉 and 〈4, 4, 5, 5, 0〉, respectively.

An oriented tree [48, p. 461] is a sequence of integers *π*_1_ *π*_2_…, such that *π*_*u*_ is the parent of node *u* and *u* is a root if *π*_*u*_ = 0. Fig 1 shows some example tree topologies and corresponding integer sequence encodings. Oriented trees provide a concise and efficient method of representing genealogies, and have been used in coalescent simulations of a spatial continuum model [49, 50]. These simulations adopted the convention that the individuals in the sample (leaf nodes) are mapped to the integers 1, …, *n*. For every internal node *u* we have *n* < *u* < 2*n* and (for a binary tree) the root is 2*n* − 1. We refer to such trees as dense because the 2*n* − 2 non-zero entries of the (binary) tree *π* occur at *u* = 1, …, 2*n* − 2. A sparse oriented tree (or more concisely, sparse tree) is an oriented tree *π* in which the leaf nodes are 1, …, *n* as before, but internal nodes can be any integer > *n*. For example, the oriented trees 〈5, 4, 4, 5, 0〉 and 〈6, 5, 5, 0, 6, 0〉 are topologically equivalent, but the former is dense and the latter sparse.

In our simulations, ancestral nodes are numbered sequentially from *n* + 1, and a new node is created when a coalescence occurs within one or more of the marginal genealogies. Note that we make a distinction between common ancestor events and coalescence events throughout. A common ancestor event occurs when two ancestors merge to form a common ancestor. If these ancestors have overlapping ancestral material, then there will also be at least one coalescence event, which is defined as a single contiguous block of sequence coalescing within a common ancestor. In Hudson’s algorithm there are many common ancestor events that do not result in coalescence, and it is important to distinguish between them.

Let the tuple (*ℓ*, *r*, *u*) define a segment carrying ancestral material. This segment represents the mapping of the half-closed genomic interval [*ℓ*, *r*) to the tree node *u*. Each ancestor *a* is defined by a set of non-overlapping segments. Initially we have *n* ancestors, each consisting of a single segment (0, *m*, *u*) for 1 ≤ *u* ≤ *n*. The only other state required by the algorithm is the time *t*, and the next node *w*; initially, *t* = 0 and *w* = *n* + 1.

Let *P* be the set of ancestors at a given time *t*. Recombination events happen at rate *ρL*/(*m* − 1) where

$$\begin{array}{c} L=\sum_{a\in P}\left(\max_{(\ell ,r,u)\in a}r-\min_{(\ell ,r,u)\in a}\ell -1\right) \end{array}$$

is the number of available ‘links’ that may be broken. (We use a fixed recombination rate here for simplicity, but an arbitrary recombination map can be incorporated without difficulty.) We choose one of the available breakpoints uniformly, and split the ancestry of the individual at that point into two recombinant ancestors. If this breakpoint is at *k*, we assign all segments with *r* ≤ *k* to one ancestor and all segments with *ℓ* ≥ *k* to the other. If there is a segment (*ℓ*, *r*, *u*) such that *ℓ* < *k* < *r*, then *k* falls within this segment and it is split such that the segment (*ℓ*, *k*, *u*) is assigned to one ancestor and (*k*, *r*, *u*) is assigned to the other.

Common ancestor events occur at rate |*P*|(|*P*| − 1). Two ancestors *a* and *b* are chosen and their ancestry merged to form their common ancestor. If their segments do not overlap, the set of ancestral segments of the common ancestor is the union of those of *a* and *b*. If segments do overlap, we have coalescence events which must be recorded. We define a coalescence event as the merging of two segments over the interval [*ℓ*, *r*) into a single ancestral segment. In general the coordinates of overlapping segments *x* and *y* will not exactly coincide, in which case we create an equivalent set of segments by subdividing into the intersections and ‘overhangs’. Suppose then that we have two exactly intersecting segments (*ℓ*, *r*, *u*) and (*ℓ*, *r*, *v*) from *a* and *b* respectively; over the interval [*ℓ*, *r*) the nodes *u* and *v* coalesce into a common ancestor, which we associate with the next available node *w*. We record this information by storing the coalescence record (*ℓ*, *r*, *w*, (*u*, *v*), *t*). As we see in the **Generating trees** section, these records provide sufficient information to later recover all marginal trees. After recording this coalescence, we then check if there are any other segments in *P* that intersect with [*ℓ*, *r*). If there are, the simulation of this region is not yet complete and we insert the segment (*ℓ*, *r*, *w*) into the ancestor of *a* and *b*. On the other hand, if there is some subset of [*ℓ*, *r*) such that there is no other segment in *P* that intersects with it, we know that the marginal tree covering this interval is complete and therefore we do not need to trace its history any further. If any other intervals overlap in *a* and *b*, we perform the same operations, and finally update the next available node by incrementing *w*. In this way, all coalescing intervals within the same ancestor map to the same node *w*, even if they are disjoint. Conversely, if two disjoint marginal trees contain the same node, we know that this is because multiple segments coalesced simultaneously within the same ancestor.

The algorithm continues generating recombination and common ancestor events at the appropriate rates until *P* is empty, and all marginal trees are complete. This interpretation of Hudson’s algorithm differs from the standard formulations [4, 5, 12] by concretely defining the representation of ancestry and by introducing the idea of coalescence records. We have omitted many important details here in the interest of brevity; see S1 Text for a detailed listing of our implementation of Hudson’s algorithm, and S2 Text for an illustration of a complete invocation of the algorithm.

There are several advantages to our sparse tree representation of ancestry. Firstly, we do not need to store partially built trees in memory, and the only state we need to maintain is the set of ancestral segments. This leads to substantial time and memory savings, since we no longer have to copy partially built trees at recombination events or update them during coalescences. We can also actively defragment the segments in memory. For example, suppose that as a result of a common ancestor event we have two segments (*ℓ*, *k*, *u*) and (*k*, *r*, *u*) in an ancestor. We can replace these segments with the equivalent segment (*ℓ*, *r*, *u*). Such defragmentation yields significant time and memory savings.

We have developed an implementation of Hudson’s algorithm called msprime based on these ideas. This package (written in C and Python) provides an ms compatible command line interface along with a Python API, and is freely available under the terms of the GNU GPL at https://pypi.python.org/pypi/msprime. The implementation uses a simple linked-list based representation of ancestral segments, and uses a binary indexed tree [51, 52] to ensure the choice of ancestral segment involved in a recombination event can be done in logarithmic time. The implementation of msprime is based on the listings for Hudson’s algorithm given in S1 Text, which should provide sufficient detail to make implementation in a variety of languages routine.

#### Performance analysis

Surprisingly little is known about the complexity of Hudson’s algorithm. We do not know, for example, what the expected maximum number of extant ancestors is, nor the distribution of ancestral material among them. The most important unknown value in terms of quantifying the complexity of the algorithm is the expected number of events that must be generated. It is sufficient to consider the recombination events as the number of common ancestor and recombination events is approximately equal [24]. Hudson’s algorithm traverses a subset of the ARG as it generates the marginal genealogies in which we are interested, and so we know that the expected number of recombination events we encounter is less than *e*^*ρ*^ [10]. This subset of the ARG is sometimes known as the ‘little’ ARG, but the relationship between the ‘big’ and little ARGs has not been well characterised.

**Fig 2:**
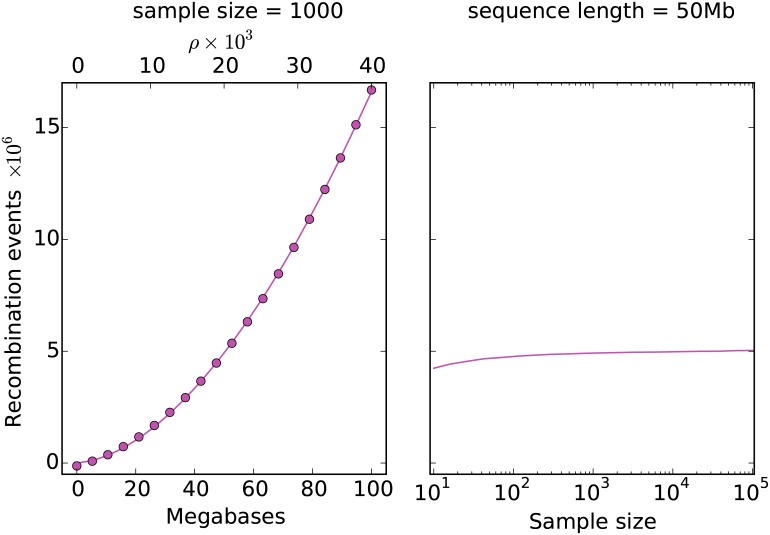
The mean number of recombination events in Hudson’s algorithm over 100 replicates for varying sequence length and sample size. In the left panel we fix *n* = 1000 and vary the sequence length. Shown in dots is a quadratic fitted to these data, which has a leading coefficient of 8.4 × 10^−3^. In the right panel we fix the sequence length at 50 megabases and vary the sample size.

Fig 2 plots the average number of recombination events generated by Hudson’s algorithm for varying sequence lengths and sample sizes. In this plot we also show the results of fitting a quadratic function to the number of recombination events as we increase the scaled recombination rate *ρ*. The fit is excellent, suggesting that the current upper bound of *e*^*ρ*^ is far too pessimistic. Wiuf and Hein [24] previously noted that the observed number of events in Hudson’s algorithm was ‘subexponential’ but did not suggest a quadratic bound. Another point to note is that the rate at which the number of events grows as we increase the sample size is extremely slow, suggesting that Hudson’s algorithm should scale well for large sample sizes.

**Fig 3:**
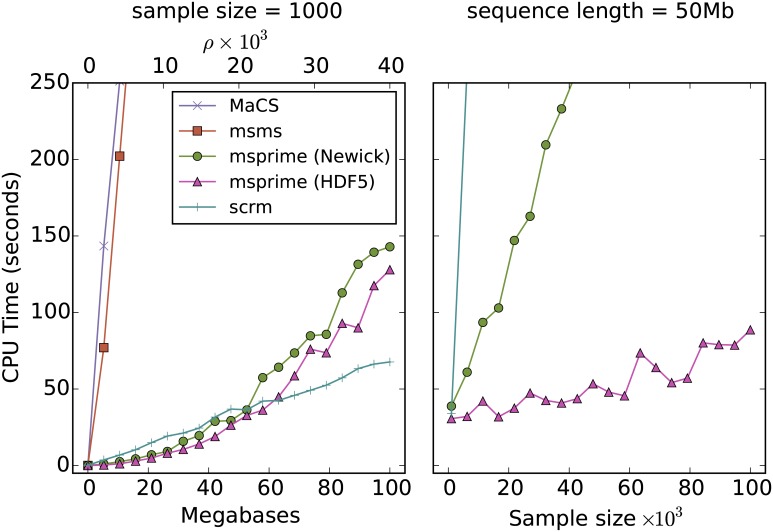
Comparison of the average running time over 100 replicates for various coalescent simulators with varying sequence length and sample size. msms [34] is the most efficient published simulator based on Hudson’s algorithm that can output genealogies. MaCS [14] is a popular SMC based simulator, and scrm [16] is the most efficient sequential simulator currently available. Both MaCS and scrm were run in SMC′ mode. Two results are shown for msprime; one outputting Newick trees and another outputting the native HDF5 based format.

These expectations are borne out well in observations of our implementation of Hudson’s algorithm in msprime. Fig 3 compares the time required to simulate coalescent trees using a number of simulation packages. As we increase the sequence length in the left-hand panel, the running time of msprime increases faster than linearly, but at quite a slow rate. msprime is faster than the SMC approximations (MaCS and scrm) until *ρ* is roughly 20000, and the difference is minor for sequence lengths greater than this. msprime is far faster than msms, the only other exact simulator in the comparison (we did not include ms in these comparisons as it was too slow and is unreliable for large sample sizes). As we increase the sample size in the right-hand panel, we can see that msprime is far faster than any other simulator. Two versions of msprime are shown in these plots: one outputting Newick trees (to ensure that the comparison with other simulators is fair), and another that outputs directly in msprime’s native format. Conversion to Newick is an expensive process, particularly for larger sample sizes. When we eliminate this bottleneck, simulation time grows at quite a slow, approximately linear rate. The memory usage of msprime is also modest, with the simulations in Fig 3 requiring less than a gigabyte of RAM. S3 Fig shows that the mean number of recombination breakpoints (i.e., the number of recombination events within ancestral material) output by all these simulators is identical, and matches Hudson and Kaplan’s prediction [6] very well, giving us some confidence in the correctness of the results. S4 Fig shows the relative performance of msprime and scrm for a small sample size, and also shows the effect of increasing the size of scrm’s sliding window.

We are often interested in the haplotypes that result from imposing a mutation process onto genealogies as well as the genealogies themselves. S1 Fig compares the time required to generate haplotypes using scrm, msprime and cosi2. Simulation times are similar in all three for a fixed sample size of 1000 and increasing sequence length. For increasing sample sizes, both cosi2 and msprime are substantially faster than scrm. However, msprime is significantly faster than cosi2 (and uses less memory; see S2 Fig), particularly when we remove the large overhead of outputting the haplotypes in text form.

Performance statistics were measured on Intel Xeon E5-2680 processors running Debian 8.2. All code required to run comparisons and generate plots is available at https://github.com/jeromekelleher/msprime-paper.

### Efficient genealogical analysis

There has been much recent interest in the problem of representing large scale genetic data in formats that facilitate efficient access and calculation of statistics [53–55]. The use of ‘succinct’ data structures, which are highly compressed but also allow for efficient queries is becoming essential: the scale of the data available to researchers is so large that naive methods simply no longer work.

Although genealogies are fundamental to biology, there has been little attention to the problem of encoding trees in a form that facilitates efficient computation. The majority of research has focused on the accurate interchange of tree structures and associated metadata. The most common format for exchanging tree data is the Newick format [56], which although ill-defined [57] has become the de-facto standard. Newick is based on the correspondence of tree structures with nested parentheses, and is a concise method of expressing tree topologies. Because of this recursive structure, specific extensions to the syntax are required to associate information with tree nodes [58, 59]. XML based formats [57, 60] are much more flexible, but tend to require substantially more storage space than Newick [57]. Various extensions to Newick have been proposed to incorporate more general graph structures [61–64], as well as a GraphML extension to encode ARGs directly [65]. Because Newick stores branch lengths rather than node times, numerical precision issues also arise when summing over many short branches [65].

General purpose Bioinformatics toolkits such as BioPerl [66] and BioPython [67] provide basic tools to import trees in the various formats. More specific tree processing libraries such as DendroPy [68], ETE [69], and APE [70] provide more sophisticated tools such as visualisation and tree comparison algorithms. None of these libraries are designed to handle large collections of correlated trees, and cannot make use of the shared structure within a sequence of correlated genealogies. The methods employed rarely scale well to trees containing hundreds of thousands of nodes.

In this section we introduce a new representation of the correlated trees output by a coalescent simulation using coalescence records. In the **Tree sequences** subsection we discuss this structure and show how it compares in practice to existing approaches in terms of storage size. Then, the **Generating trees** subsection presents an algorithm to sequentially generate the marginal genealogies from a tree sequence, which we compare with existing Newick-based methods. Finally, in the **Counting leaves** subsection we show how the algorithm to sequentially visit trees can be extended to efficiently maintain the counts of leaves from a specific subset, and show how this can be applied in a calculation commonly used in genome wide association studies.

#### Tree sequences

**Fig 4:**
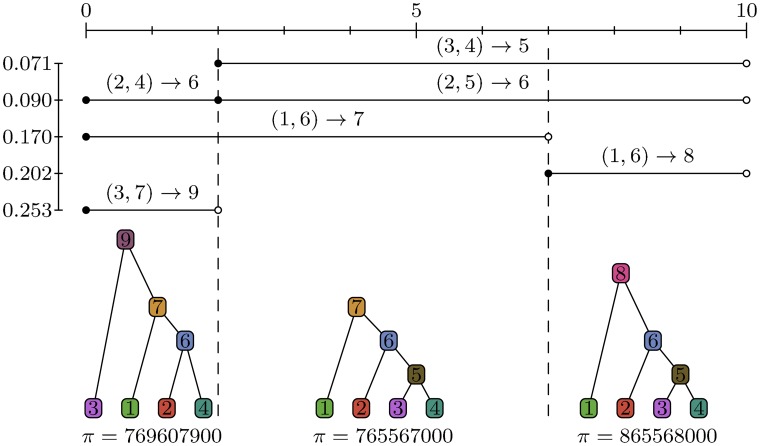
Coalescence records and corresponding marginal trees. The *x*-axis represents genomic coordinates, and *y*-axis represents time (with the present at the top). Each line segment in the top section of the figure represents a coalescence record; e.g., the first segment corresponds to the coalescence record (2, 10, 5, (3, 4), 0.071). The lower section of the figure shows the corresponding trees in pictorial and sparse tree form. We have omitted commas and brackets from this sequence representation for compactness.

As described earlier, the output of our formulation of Hudson’s algorithm is a list of coalescence records. Each coalescence record is a tuple (*ℓ*, *r*, *u*, *c*, *t*) describing the coalescence of a list of child nodes *c* into the parent *u* at time *t* over the half-closed genomic interval [*ℓ*, *r*). (Because only binary trees are possible in the standard coalescent, we assume the child node list *c* is a 2-tuple (*c*_1_, *c*_2_) throughout. However, arbitrary numbers of children can be accommodated without difficulty to support common ancestor events in which more than two lineages merge [71–74]) We refer to this set of records as a *tree sequence*, as it is a compact encoding of the set of correlated trees representing the genealogies of a sample. Fig 4 shows an illustration of the tree sequence output by an example simulation (see S2 Text for a full trace of this simulation).

The tree sequence provides a concise method of representing the correlated genealogies generated by coalescent simulations because it stores node assignments shared across adjacent trees exactly once. Consider node 7 in Fig 4. This node is shared in the first two marginal trees, and in both cases it has two children, 1 and 6. Even though the node spans two marginal trees, the node assignment is represented in one coalescence record (0, 7, 7, (1, 6), 0.170). Importantly, this holds true even though the subtree beneath 6 is different in these trees. Thus, any assignment of a pair of children to a given parent that is shared across adjacent trees will be represented by exactly one coalescence record.

Coalescence records provide a full history of the coalescence events that occurred in our simulation. (Recall that we distinguish between common ancestor events, which may or may not result in marginal coalescences, and coalescence events which are defined as a single contiguous block of genome merging within a common ancestor.) The effects of recombination events are also stored indirectly in this representation in the form of the left and right coordinate of each record. For every distinct coordinate between 0 and *m*, there must have been at least one recombination event that occurred at that breakpoint. However, there is no direct information about the times of these recombination events, and many recombinations will happen that leave no trace in the set of coalescence records. For example, if we have a recombination event that splits the ancestry of a given lineage, and this is immediately followed by a common ancestor event involving these two lineages, there will be no record of this pair of events.

On the other hand, if we consider the records in order of their left and right coordinates we can also see them as defining the way in which we transform the marginal genealogies as we move across a chromosome. Because many adjacent sites may share the same genealogy, we need only consider the coordinates of our records in order to recover the distinct genealogies and the coordinate ranges over which they are defined. To obtain the marginal tree covering the interval [0,2), for example, we simply find all records with left coordinate equal to 0 and apply these to the empty sparse tree *π*. To subsequently obtain the tree corresponding to the interval [2, 7) we first remove the records that do not apply over this interval, which must have right coordinate equal to 2. In the example, this corresponds to removing the assignments (2, 4) → 6 and (3, 7) → 9. Having removed the ‘stale’ records that do not cover the current interval, we must now apply the new records that have left coordinate 2. In this case, we have two node assignments (3, 4) → 5 and (2, 5) → 6, and applying these changes to the current tree completes the transformation of the first marginal tree into the second.

There is an important point here. As we moved from left-to-right across the simulated chromosome we transitioned from one marginal tree to the next by removing and applying only two records. Crucially, modifying the nodes that were affected by this transition did not result in a relabelling of any nodes that were not affected. As Wiuf and Hein [24,75] showed, the effect of a recombination at a given point in the sequence is to cut the branch above some node in the tree to the left of this point, and reattach it within another branch. This process is known as a subtree-prune-and-regraft [76, 77] and requires a maximum of three records to express in our tree sequence formulation.

**Fig 5:**
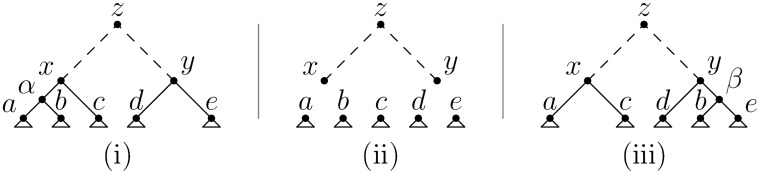
A prune and regraft not involving the root requires three records. (i) We begin with two subtrees rooted at *x* and *y*, and we wish to prune the subtree rooted at *b* and graft it in the branch joining *e* to *y*. (ii) We remove the assignments (*a*, *b*) → *α*, (*α*, *c*) → *x* and (*d*, *e*) → *y*. After this operation, the subtrees *a*, …, *e* are disconnected from the main tree. The main trunk the tree rooted at *z* is unaffected, as are the subtrees below *a*, …, *e*. (iii) We add the records (*a*, *c*) → *x*, (*b*, *e*) → *β* and (*d*, *β*) → *y*, completing the transition.

Prune-and-regraft operations that do not affect the root require three records, as illustrated in Fig 5. Two other possibilities exist for how the current tree can be edited as we move along the sequence. The first case is when we have a prune and regraft that involves a change in root node; this requires only two records and is illustrated in the first transition in Fig 4. The other case that can arise from a single recombination event is a simple root change in which the only difference between the adjacent trees is the time of the MRCA. This requires one record, and is illustrated in the second transition in Fig 4. These three possibilities are closely related to the three classes of subtree-prune-and-regraft identified by Song [76, 77].

Knowing the maximum number of records arising from a single recombination event provides us with a useful bound on the expected number of records in a tree sequence. Because the expected number of recombination events within ancestral material is approximately *ρ* log* n* [6, 24] we know that the expected number of tree transitions is *ρ* log* n*. The number of records we require for these tree transitions is then clearly ≤ 3*ρ* log* n*. We also require *n* − 1 records to describe the first tree in the sequence, and so the total number of records is ≤ *n* + 3*ρ* log* n* − 1.

Storing a tree sequence as a set of coalescence records therefore requires *O*(*n* + *ρ* log* n*) space, whereas any representation that stores each tree separately (such as Newick) must require *O*(*nρ* log* n*) space. This difference is substantial in practice. As an example of a practical simulation of the sort currently being undertaken, we repeated the simulation run by Layer et al. [54], in which we simulate a 100 megabase region with a recombination rate of 10^−3^ per base per 4*N*_*e*_ generations for a sample of 100,000 individuals. This simulation required approximately 6 minutes and 850MB of RAM to run using msprime; the original simulation reportedly required over 4 weeks using MaCS on similar hardware.

Outputting the results as coalescence records in a simple tab-delimited text format resulted in a 173MB file (52MB when gzip compressed). In contrast, writing the trees out in Newick form required around 3.5TB of space. Because plain text is a poor format for storing structured numerical data [78], msprime provides a tree sequence storage file format based of the HDF5 standard [79]. Using this storage format, the file size is reduced to 88MB (41MB using the transparent zlib compression provided by the HDF5 library).

To compare the efficiency of storing correlated trees as coalescence records with the TreeZip compression algorithm [80] we output the first 1000 trees in Newick format, resulting in a 3.2GB text file (1.1GB gzip compressed). The TreeZip compression algorithm required 10 hours to run and resulted in an 882MB file (83MB gzip compressed). Unfortunately, it was not feasible to run TreeZip on all 3.5TB of the Newick data, but we can see that with only around 0.1% of the input data, the compressed representation is already larger than the simple text output of the entire tree sequence when expressed as coalescence records.

Associating mutation information with a tree sequence is straightforward. For example, to represent a mutation that occurs on the branch that joins node 7 to node 9 at site 1 in Fig 4, we simply record the tuple (7, 1). (Infinite sites mutations can be readily accommodated by assuming that the coordinate space is continuous rather than discrete.) Because only the associated node and position of each mutation needs to be stored, this results in a very concise representation of the full genealogical history and mutational state of a sample. Repeating the simulation above with a scaled mutation rate of 10^−3^ per unit of sequence length per 4*N*_*e*_ generations resulted in 1.2 million infinite sites mutations. The total size of the HDF5 representation of the tree sequence and mutations was 102MB (49MB using HDF5’s zlib compression). In contrast, the text-based haplotype strings consumed 113GB (9.7GB gzip compressed). Converting to text haplotypes required roughly 9 minutes and 14GB of RAM.

The PBWT [53] represents binary haplotype data in a format that is both highly compressed and enables efficient pattern matching algorithms. We converted the mutation data above into PBWT form, which required 22MB of storage. Thus, the PBWT is a more compact representation of a set of haplotypes than the tree sequence. However, the PBWT does not contain any genealogical data, and therefore contains less information than the tree sequence.

#### Generating trees

Coalescence records provide a very compact means of encoding correlated genealogies. Compressed representations of data usually come at the cost of increased decompression effort when we wish to access the information. In contrast, we can recover the marginal trees from a set of coalescence records orders of magnitude more quickly than is possible using existing methods. In this section we define the basic algorithm required to sequentially generate these marginal genealogies.

For algorithms involving tree sequences it is useful to regard the set of coalescence records as a table and to index the columns independently (see S2 Text for the table corresponding to Fig 4). Therefore define a tree sequence *T* as a tuple of vectors *T* = (**l**, **r**, **u**, **c**, **t**), such that for each index 1 ≤ *j* ≤ *M*, (**l**_*j*_, **r**_*j*_, **u**_*j*_, **c**_*j*_, **t**_*j*_) corresponds to one coalescence record output by Hudson’s algorithm, and there are *M* records in total. It is also useful to impose an ordering among the children at a node, and so we assert that **c**_*j*,1_ < **c**_*j*,2_ for all 1 ≤ *j* ≤ *M*.

If we wish to obtain the tree for a given site *x* we simply find the *n* − 1 records that intersect with this point and build the tree by applying these records. We begin by setting *π*_*j*_ ← 0 for 1 ≤ *j* ≤ max(**u**), and then set *π*_**c**_*j*,1__ ← **u**_*j*_ and *π*_**c**_*j*,2__ ← **u**_*j*_ for all *j* such that **l**_*j*_ ≤ *x* < **r**_*j*_. Spatial indexing structures such as the segment tree [81] allow us to find all *k* segments out of a set of *N* that intersect with a given point in *O*(*k* + log* N*) time. Therefore, since the expected number of records is *O*(*n* + *ρ* log* n*) as shown in the previous subsection, the overall complexity of generating a single tree is *O*(*n* + log(*n* + *ρ* log* n*)).

A common requirement is to sequentially visit all trees in a tree sequence in left-to-right order. One possible way to do this would be to find all of the distinct left coordinates in the **l** vector and apply the process outlined above. However, adjacent trees are highly correlated and share much of their structure, and so this approach would be quite wasteful. A more efficient approach is given in Algorithm T below. For this algorithm we require two ‘index vectors’ I and O which give the indexes of the records in the order in which they are inserted and removed, respectively. Records are applied in order of nondecreasing left coordinate and increasing time, and records are removed in nondecreasing order of right coordinate and decreasing time. That is, for every pair of indexes *j* and *k* such that 1 ≤ *j* < *k* ≤ *M* we have either $l_{I_{j}}<l_{I_{k}}$ or $l_{I_{j}}=l_{I_{k}}$ and $t_{I_{j}}<t_{I_{k}}$; and similarly, either $r_{O_{j}}<r_{O_{k}}$ or $r_{O_{j}}=r_{O_{k}}$ and $t_{O_{j}}>t_{O_{k}}$. We assume that these index vectors have been pre-calculated below.

**Algorithm T.** (*Generate trees*). Sequentially visit the sparse trees *π* in a tree sequence *T* = (**l**,**r**,**u**,**c**,**t**) with *M* records.

**T1.** [Initialisation.] Set *π*_*j*_ ← 0 for 1 ≤ *j* ≤ max(**u**). Then set *j* ← 1, *k* ← 1 and *x* ← 0.

**T2.** [Insert record.] Set $h\leftarrow I_{j}$, *π*_**c**_*h*,1__ ← *π*_**c**_*h*,2__ ← **u**_*h*_, and *j* ← *j* + 1. If *j* ≤ *M* and $l_{I_{j}}=x$, go to T2.

**T3.** [Visit tree.] Visit the sparse tree *π* starting at site *x*. If *j* > *M* terminate the algorithm. Otherwise, set $x\leftarrow l_{I_{j}}$.

**T4.** [Remove record.] Set $h\leftarrow O_{k}$, *π*_**c**_*h*,1__ ← *π*_**c**_*h*,2__ ← 0 and *k* ← *k* + 1. Then, if $r_{O_{k}}=x$ go to T4; otherwise, go to T2.

Algorithm T sequentially generates all marginal trees in a tree sequence by first applying records to the sparse tree *π* in step T2 for a given left coordinate. Once this is complete, the tree is made available to client code by ‘visiting’ it [48, p.281] in T3. After the user has finished processing the current tree, we prepare to move to the next tree by removing all stale records in T4, and then return to T2. The algorithm is very efficient. Because each record is considered exactly once in step T2 and at most once in step T4 the total time required by the algorithm is *O*(*n* + *ρ* log* n*). To illustrate this efficiency, we consider the time required to iterate over the trees produced by the large example simulation used throughout this section. Reading in the full tree sequence in msprime’s native HDF5 based format and iterating over all 1.1 million trees using the Python API required approximately 3 seconds. In contrast, using the BioPython [67] version 1.64 Newick parser required around 3 seconds *per tree*, leading to an estimated 38 days to iterate over all trees. Similarly, ETE [69] version 2.3.9 required 4.5 seconds per tree, and DendroPy [68] version 4.0.2 required around 14 seconds per tree. Comparing Python Newick parsers to msprime may be somewhat misleading, since the majority of msprime’s tree processing code is written in C. However, APE [70] version 3.1, which uses a Newick parser written in C, also required around 7 seconds per tree. Thus, using msprime’s API we can iterate over this set of trees more than a *million times* faster than any of these alternatives.

Algorithm T generates only the sparse tree *π* mapping each node to its parent. It is easy to extend this algorithm to include information about the node times, children, start and end coordinates and other information. We have also assumed binary trees here, but it is trivial to extend the algorithm to work with more general trees. When computing statistics across the tree sequence it is often useful to know the specific differences between adjacent trees, as this often allows us to avoid examining the entire tree. This information is directly available in Algorithm T. The tree iteration code in msprime’s Python API makes all of this information available, facilitating easy tree traversal in both top-down and bottom-up fashion.

#### Counting leaves

The previous subsection provides an algorithm to efficiently visit all marginal genealogies in a tree sequence. This algorithm can be easily augmented to maintain summaries of tree properties as we sweep across the sequence. As an example of this, we show how to augment Algorithm T to maintain the counts of the number of leaves from a specific set that are below each internal node. More precisely, given some subset *S* of our sample, we maintain a vector *β* such that for any node *u*, *β*_*u*_ is the number of leaves below *u* that belong to the set *S*. This allows us to quickly calculate allele frequencies: since each mutation is associated with a particular node *u*, *β*_*u*_/|*S*| is the frequency of the mutation within *S*. Calculating allele frequencies within specific subsets of the sample has many applications, for example calculating summary statistics such as *F*_*ST*_ [82], and association tests in genome wide association studies [83].

Suppose we have a tree sequence *T* and we wish to generate the sparse trees *π* as before. We now also wish to generate the vector *β*, such that *β*_*u*_ gives the number of leaf nodes in the subtree rooted at *u* that are in the set *S* ⊆ {1, …, *n*}. We assume that the index vectors I and O have been precomputed, as before.

**Algorithm L.** (*Count leaves*). Generate the sparse trees *π* and leaf counts *β* for a tree sequence *T* = (**l**,**r**,**u**,**c**,**t**) with *M* records and set of leaves *S*.

**L1.** [Initialisation.] Set *π*_*j*_ ← *β*_*j*_ ← 0 for 1 ≤ *j* ≤ max(**u**). Set *β*_*j*_ ← 1 for each *j* ∈ *S*. Then set *j* ← 1, *k* ← 1 and *x* ← 0.

**L2.** [Insert record.] Set $h\leftarrow I_{j}$, *π*_**c**_*h*,1__ ← *π*_**c**_*h*,2__ ← **u**_*h*_, *b* ← *β*_**c**_*h*,1__+*β*_**c**_*h*,2__ and *j* ← *j* + 1.

**L3.** [Increment leaf counts.] Set *v* ← **u**_*h*_. Then, while *v* ≠ 0, set *β*_*v*_ ← *β*_*v*_ + *b* and *v* ← *π*_*v*_. Afterwards, if *j* ≤ *M* and $l_{I_{j}}=x$, go to L2.

**L4.** [Visit tree.] Visit (*π*, *β*). If *j* > *M* terminate the algorithm; otherwise, set $x\leftarrow l_{I_{j}}$.

**L5.** [Remove record.] Set $h\leftarrow O_{k}$, *π*_**c**_*h*,1__ ← *π*_**c**_*h*,2__ ← 0, *b* ← *β*_**c**_*h*,1__+*β*_**c**_*h*,2__ and *k* ← *k* + 1.

**L6.** [Decrement leaf counts.] Set *v* ← **u**_*h*_. Then, while *v* ≠ 0, set *β*_*v*_ ← *β*_*v*_ − *b* and *v* ← *π*_*v*_. Afterwards, if $r_{O_{k}}=x$, go to L5; otherwise, go to L2.

Algorithm L works in the same manner as Algorithm T: for each tree transition, we remove the stale records that no longer apply to the genomic interval currently under consideration, and apply all new records that begin at location *x*. We update the sparse tree *π* by applying a record in step L2, and then update the leaf count *β* to account for this new node assignment. In step L3 we propagate the corresponding leaf count gain up to the root, before returning to L2 if necessary. Once we have applied all of the inbound records we then visit the tree by making *π* and *β* available to the user in L4. Then, if any more trees remain, we move on by removing the outbound records in steps L5 and L6, updating *β* to account for the corresponding loss in leaf counts. The correctness of the algorithm depends on the ordering of the index vectors I and O. Records are always inserted in increasing order of time, and always removed in decreasing order of time within a tree transition. Therefore, for any record in which subtrees rooted at *c*_1_ and *c*_2_ become the children of *u*, we are guaranteed that these subtrees are complete and that *β*_*c*_1__ and *β*_*c*_2__ are correct. Removing outbound records in reverse order of time similarly guarantees that the leaf counts within the disconnected subtrees that we create are maintained correctly.

Algorithm L clearly examines each record at most once in steps L2 and L5. Steps L3 and L6 contain loops to propagate leaf counts up the tree, and are therefore not constant time operations. Since coalescent genealogies are asymptotically balanced [84], the expected height of a tree (in terms of the number of nodes) is log_2_ *n*. Therefore, the cost of steps L3 and L6 is *O*(log_2_ *n*) per record, leading to a log_2_ *n* extra cost over Algorithm T. In practical terms, this extra cost is negligible. For example, msprime automatically maintains counts for all leaves (and optionally can maintain counts for specific subsets) when doing all tree transitions. The 3 second time quoted above required to iterate over all 1.1 million trees in the large simulation example includes the cost of maintaining counts for all 10^5^ leaves at all internal nodes. To demonstrate this efficiency, we ran a simple genome wide association test, where we split the sample into 50,000 cases and controls. One of the most powerful and popular applications for running such association tests is plink [85]. After converting the simulated data to a 29G BED file, the stable version of plink (1.07) required 176 minutes to run a simple association test. The development version of plink (1.9) required 54 seconds. Using msprime’s Python API, the same odds-ratio test required around 10 seconds.

## Results and Discussion

The primary contribution of this paper is to introduce a new encoding for the correlated trees resulting from simulations of the coalescent with recombination. This encoding follows on from previous work in which trees are encoded as integer vectors [49, 50], but makes the crucial change that tree vectors are sparse. Using this encoding, the effects of each coalescence event are stored as simple fixed-size records that provide sufficient information to recover all marginal genealogies after the simulation has completed. This approach leads to very large gains in simulation performance over classical simulators such as ms, so that the exact simulation of genealogies for the coalescent with recombination over chromosome scales is feasible for the first time. We have presented an implementation based on the sparse tree encoding called msprime, which is faster than all other simulators for large sample sizes. This simulator supports the full discrete population structure and demographic event model provided by ms along with variable recombination rates. We plan to include populations evolving in continuous space [86–88] and gene conversion [89] in subsequent releases.

Coalescence records also lead to an extremely compact storage format that is several orders of magnitude smaller than the most compact method currently available. Despite this very high level of compression, accessing the genealogical data is very efficient. In an example with 100,000 samples, we saw a roughly 40,000-fold reduction in file size over the Newick tree encoding, and a greater than million-fold decrease in the time required to iterate over the genealogies compared to several popular libraries. This efficiency is gained through very simple algorithms that we have stated rigorously and unambiguously, and also analysed in terms of their computational complexity. Being able to process such large sample sizes is not an idle curiosity; on the contrary, we have a pressing need to work with such datasets. We envisage three immediate uses for our work.

Firstly, sequencing projects are being conducted on an unprecedented scale [90–95], and the storage and analysis of these data pose serious computational challenges. Sophisticated new methods are being developed to organise and analyse information on this immense scale [53–55]. Developers have struggled to generate simulated data on a similar scale [53, 54], as present day simulators perform poorly on these huge sample sizes. Using msprime, the time required to generate genome scale data for hundreds of thousands of samples is reduced from weeks to minutes.

Secondly, prospective studies such as UK Biobank [96, 97] are collecting genetic and high-dimensional phenotypic data for hundreds of thousands of samples. The key statistical method to interrogate such data is the genome wide association study (GWAS) [98], and large sample size has been identified as the single most important factor in determining the power of these studies [83]. Simulation plays a key role in GWAS, and typically proceeds by superimposing the disease model of interest on haplotypes obtained via various methods [99]. Because the accurate modelling of linkage disequilibrium is essential in disease genetics [100], recombination must be incorporated. Resampling methods [83, 101–103] generate simulated haplotypes based on an existing reference panel, and provide a good match to observed linkage patterns. However, there is some bias associated with this process, and there are statistical difficulties when the size of the sample required is larger than the reference panel. Other methods obtain simulated haplotypes from population genetics models via forwards-in-time [104, 105] or coalescent [106, 107] simulations. None of these methods can efficiently handle the huge sample sizes required, however. A simulator for high dimensional phenotype data based on msprime could alleviate these performance issues and be a key application for the library.

Thirdly, today’s large sample sizes provide us with an unprecedented opportunity to understand the history and geographic structure of our species. Aside from its intrinsic interest, correctly accounting for population stratification is critical for the interpretation of association studies [108, 109], particularly for rare variants [110, 111]. Researchers are seeking to understand fine scale population structure using methods based on principal component analysis [112], admixture fractions [113–115], length of haplotype blocks [116–118] and allele frequencies [119]. To date, it has been challenging to assess the accuracy of these methods, as simulations struggle to match the required sequence lengths and sample sizes. Furthermore, methods based on the SMC approximation [17, 18] have been tested using SMC simulations out of necessity, making it difficult to assess the impact of the approximation on accuracy. Simulations of the exact coalescent with recombination at chromosome scales for large sample sizes and arbitrary demographies will be an invaluable tool for developers of such methods.

As we have demonstrated, the tree sequence structure leads to very efficient algorithms, and allows us to encode simulated data very compactly. We would also wish to encode biological data in this structure so that we can apply these algorithms to analyse real data. However, to do this we must estimate a tree sequence from data, which is a non-trivial task. Nonetheless, there has been much work in this area [120] with several heuristic [121] and more principled approaches that may be adopted [19, 122]. Using the PBWT [53] to find long haplotypes (which will usually correspond to long records) seems like a particularly promising avenue.

Finally, an interesting issue arises when we consider the problem of inferring a tree sequence from data. Suppose we have observed a set of haplotypes resulting from a coalescent simulation with infinite sites mutations occurring at a very high rate. Under these conditions, the underlying tree sequence can be recovered exactly from the data, but the corresponding ARG (i.e., the specific realisation of the ARG that was traversed by Hudson’s algorithm) cannot. For example, a recombination may have occurred during the simulation that was immediately followed by a common ancestor event involving the same lineages. These nodes in the ARG can have no effect on the data, and are therefore unobservable. To put this in another way, there is no observable information in an ARG that is not in a tree sequence. Given this representational sufficiency and the storage and processing efficiencies demonstrated in this article, we would argue that a tree sequence is a more natural and powerful representation of observed genetic variation than an ARG.

## Supporting Information

S1 Text Detailed listing of Hudson’s algorithm. (PDF)

S2 Text Illustration of Hudson’s algorithm. (PDF)

S1 Fig Comparisons of the running times for various coalescent simulators to generate mutations for varying sequence length and sample size. We use a scaled mutation rate of *θ* = 4*N*_*e*_*μ* = 0.0004.
(PDF)

S2 Fig The corresponding maximum memory usages for the simulators in S1 Fig. (PDF)

S3 Fig The mean number of recombination breakpoints for the simulations in Fig 3 along with the theoretical prediction (black line). This plot shows that the number of recombination events within ancestral material for these simulations is identical for all simulators and agrees very well with the theoretical value of *ρH*_*n* − 1_, where *H*_*n*_ is the *n*th Harmonic number.
(PDF)

S4 Fig Comparison of simulation times with msprime and scrm for a sample size of *n* = 20 with increasing sequence length. Several different approximation levels are shown for scrm using the -l option. The -l 500r option is described as a conservative value giving very good accuracy, and -l 100r is recommended as a good compromise between running time and accuracy.
(PDF)
