# Supplementary material for: Efficient Coalescent Simulation and Genealogical Analysis for Large Sample Sizes
Source: PLoS Comput Biol. 2016 May 4;12(5):e1004842. doi: 10.1371/journal.pcbi.1004842 (PMC4856371; doi:10.1371/journal.pcbi.1004842)
Supplement: S1 Text — (PDF) [file pcbi.1004842.s001.pdf]

Detailed listing of Hudson's Algorithm

In this section we provide a detailed description of our implementation of Hudson's algorithm. First, we require some notation. Let $\mathcal{R}_\Delta(\xi_1, \dots, \xi_k)$ define a single independent sample from a random variable with distribution Δ and parameters $\xi_1, \dots, \xi_k$. (Note that each instance of $\mathcal{R}_\Delta(\xi_1, \dots, \xi_k)$ within an algorithm listing represents an *independent* random sample from the specified distribution.) Using this notation, we define $\mathcal{R}_U(A)$ to be an element of the set A chosen uniformly at random, and $\mathcal{R}_E(\lambda)$ as a sample from an exponentially distributed random variable with rate λ. We use a simple linked list representation of ancestral segments such that for a segment z, $\text{prev}(z)$ denotes the previous segment to z in the linked list, and similarly $\text{next}(z)$ denotes the next segment. Let Λ denote a special segment indicating the end of a chain (the null reference is convenient for this purpose in many languages). Let $z \leftarrow \text{Segment}(\ell, r, u, x, y)$ denote a newly allocated segment such that $\text{left}(z) = \ell$, $\text{right}(z) = r$, $\text{node}(z) = u$, $\text{prev}(z) = x$ and $\text{next}(z) = y$. We sometimes omit the last two parameters for convenience; in this case, they are implicitly defined as Λ, and therefore $\text{Segment}(\ell, r, u) = \text{Segment}(\ell, r, u, \Lambda, \Lambda)$. Each element of a linked list of these segments corresponds to a contiguous block of ancestry in which we map the node u to the half-closed interval $[\ell, r)$.

During recombination events we choose a breakpoint randomly and split the ancestral material within an ancestor at that point. We model these breakpoints as 'links' between adjacent sites. We use a binary indexed tree (Fenwick, 1994, 1995) L to track the cumulative number of links subtended by each extant segment (segments are ordered arbitrarily in this cumulative sum over the segments in extant ancestors). A segment x subtends $\text{right}(x) - \text{left}(x) - 1$ links if it is the first in a chain; if it is not, it subtends $\text{right}(x) - \text{right}(\text{prev}(x))$. That is, a segment is associated with all the links that fall both within the interval it covers and also with the links that fall in the interval between it and its predecessor. To set the number of links mapped to a segment x to v, we use the notation $L_x \leftarrow v$. To find the total number of links subtended by all segments, we use $\text{total}(L)$, and to obtain the cumulative number of links subtended by segment x, we use $\text{total}(L, x)$. Finally, $\text{find}(L, v)$ returns the last segment whose cumulative sum is $\leq v$. Using these tools we can randomly choose a link and find the segment that subtends it in logarithmic time.

Termination of Hudson's algorithm works by a gradual process of removing segments in which the MRCA has been reached. We implement this by maintaining a map S that counts the number of extant segments intersecting with a given interval. We use a balanced binary tree (Knuth, 1998, §6.2.3) to store this map. To assign a value v to key k, we write $S_k \leftarrow v$. The data structure supports two further operations: $\text{search}(S, k)$ returns the largest key $\leq k$, and $\text{nextkey}(S, k)$ returns the smallest key $> k$. For each key k, S_k counts the number of extant segments in the interval $[k, \text{nextkey}(S, k))$. As the simulation proceeds we update this map to account for coalescences that occur, inserting keys and decrementing the counts as necessary.

Algorithm H. (*Hudson's algorithm*). Simulate the coalescent with recombination for a sample of n individuals on a sequence of m sites with recombination at rate r per generation between adjacent sites.

H1. [Initialisation.] Set $P \leftarrow \emptyset$, $C \leftarrow \emptyset$, $S \leftarrow \text{BalancedBinaryTree}()$ and $L \leftarrow \text{BinaryIndexedTree}()$. Then, for $1 \leq j \leq n$, set $x \leftarrow \text{Segment}(0, m, j)$, $L_x \leftarrow m - 1$ and $P \leftarrow P \cup \{x\}$. Finally, set $S_0 \leftarrow n$, $S_m \leftarrow -1$, $w \leftarrow n + 1$ and $t \leftarrow 0$.

H2. [Event.] Set $\lambda_r \leftarrow r\,\text{total}(L)$, $\lambda \leftarrow \lambda_r + |P|\,(|P|-1)$, and set $t \leftarrow t + \mathcal{R}_E(\lambda)$. If $\mathcal{R}_U([0,1)) < \lambda_r/\lambda$, invoke Algorithm R; otherwise, invoke Algorithm C.

H3. [Loop.] If $|P| \neq 0$ go to H2 .

The basic structure of Hudson's algorithm is very simple. We begin in H1 by allocating the set P to represent the extant ancestors and C to store our coalescence records. We also allocate the balanced binary tree S and the binary indexed tree L as discussed above. We then allocate a segment x covering the interval $[0, m)$, that points to node j for each individual $1 \leq j \leq n$ in the sample, record that this segment subtends $m - 1$ links and then insert it into the set of ancestors P. Afterwards, we initialise the map S by setting $S_0 \leftarrow n$ and $S_m \leftarrow -1$ (stating that the number of extant segments in the interval $[0, m)$ is n), set the next available node w to $n + 1$ and our clock t to zero.

In H2, we calculate the current rate of recombination and common ancestor events, and increment t accordingly. We then choose the type of the next event and invoke either Algorithm R or Algorithm C. Once the appropriate subroutine has completed, we move on to H3, where we either terminate or loop back to H2. Upon termination, C contains the set of coalescence records that defines the output of the algorithm.

Algorithm R implements a single recombination event by choosing a link uniformly and breaking it, resulting in a new individual being added to the set of extant ancestors. There are two possibilities for this link: it is either between two segments or within a segment, and these possibilities are dealt with separately in steps R2 and R3, respectively. In either case, z points to the head of the segment chain representing the new individual, which is inserted into P in step R4.

Algorithm R. (*Recombination event*). Choose a link uniformly and break it, resulting in one extra individual in the set of extant ancestors.

R1. [Choose link.] Set $h \leftarrow \mathcal{R}_U(\{1, \ldots, \text{total}(L)\})$, $y \leftarrow \text{find}(L, h)$, $k \leftarrow \text{right}(y) - \text{total}(L, y) + h - 1$ and $x \leftarrow \text{prev}(y)$. Then, if $\text{left}(y) < k$ go to R3.

R2. [Break between segments.] Set $\text{next}(x) \leftarrow \Lambda$, $\text{prev}(y) \leftarrow \Lambda$, $z \leftarrow y$ and go to R4.

R3. [Break within segment.] Set $z \leftarrow \text{Segment}(k, \text{right}(y), \text{node}(y), \Lambda, \text{next}(y))$. Then, if $\text{next}(y) \neq \Lambda$, set $\text{prev}(\text{next}(y)) \leftarrow z$. Afterwards, set $\text{next}(y) \leftarrow \Lambda$, $\text{right}(y) \leftarrow k$ and $L_y \leftarrow L_y + k - \text{right}(z)$.

R4. [Update population] Set $L_z \leftarrow \text{right}(z) - \text{left}(z) - 1$ and $P \leftarrow P \cup \{z\}$.

The algorithm begins in step $R1$ by choosing a link h uniformly from the total(L) that are currently being tracked. We then find the segment y that subtends this link using the binary indexed tree `find` function. Once we have found the segment in question, we then calculate the corresponding breakpoint k, so that we can determine whether link h falls within y or between y and its predecessor x. Thus, if the breakpoint $k > \text{left}(y)$, we go to R3, and otherwise proceed to step R2.

Step R2 is very straightforward. Because the breakpoint k is between the two segments x and y, we must simply break the forward and reverse links in the segment chain between them. After breaking these links, we now have an independent segment chain starting with z, which represents the new individual to be added to the set of ancestors. On the other hand, if the breakpoint k falls within y, we must split this segment in step R3 such that the ancestral material from left(y) to k remains assigned to the current individual and the remainder is assigned to the new individual z. We must also update the number of links subtended by the segment y, which has $\text{right}(z) - k$ fewer links as a result of this operation. Finally, step R4 inserts the segment z into the set of ancestors, since this is the first segment in the new individual. However, we must also update the information about the number of links subtended by this segment. Since z is the head of a new segment chain, there is no previous segment, and the number of links it subtends is $\text{right}(z) - \text{left}(z) - 1$. After this, we complete the recombination event, returning to Algorithm H.

Algorithm C implements a single common ancestor event, where we choose two individuals randomly and merge their ancestral segment chains. If these two ancestors have overlapping segments we record the corresponding coalescence events. When a coalescence occurs, we decrement the number of extant segments in the corresponding interval by updating S. When this value is reduced to 1, we discard the corresponding segment since it can have no further effect on the genealogies we are interested in. Thus, the algorithm always removes two individuals from the set of ancestors P, but may reinsert zero or one, depending on whether any ancestral segments remain after merging. By this process the size of P is eventually reduced to zero and Hudson's algorithm is complete.

Algorithm C. (*Common ancestor event*). Choose two ancestors uniformly and merge their segments, recording any coalescences that occur as a consequence.

C1. [Choose ancestors.] Set $x \leftarrow \mathcal{R}_U(P)$, $y \leftarrow \mathcal{R}_U(P \setminus \{x\})$. Then, set $P \leftarrow P \setminus \{x, y\}$, $z \leftarrow \Lambda$ and $\mathfrak{c} \leftarrow 0$.

C2. [Loop head] If $x = \Lambda$ and $y = \Lambda$, terminate the algorithm. Set $\alpha \leftarrow \Lambda$. If $x \neq \Lambda$ and $y \neq \Lambda$ go to C3. Otherwise, if $x \neq \Lambda$ set $\alpha \leftarrow x$ and set $x \leftarrow \Lambda$. If $y \neq \Lambda$ set $\alpha \leftarrow y$ and set $y \leftarrow \Lambda$. Go to C8.

C3. [Choose case] If $\text{left}(y) < \text{left}(x)$, set $\beta \leftarrow x$, $x \leftarrow y$ and $y \leftarrow \beta$. Then, if $\text{right}(x) \leq \text{left}(y)$, set $\alpha \leftarrow x$, $x \leftarrow \text{next}(x)$, $\text{next}(\alpha) \leftarrow \Lambda$ and go to C8; otherwise, if $\text{left}(x) \neq \text{left}(y)$ set $\alpha \leftarrow \text{Segment}(\text{left}(x), \text{left}(y), \text{node}(x))$, $\text{left}(x) \leftarrow \text{left}(y)$ and go to C8.

C4. [Coalescence] If $\mathfrak{c} = 0$, set $\mathfrak{c} \leftarrow 1$ and $w \leftarrow w+1$. Afterwards, set $u \leftarrow w-1$, $\ell \leftarrow \text{left}(x)$ and $r^* \leftarrow \min(\text{right}(x), \text{right}(y))$. If $\ell \notin S$, set $j \leftarrow \text{search}(S, \ell)$ and $S_\ell \leftarrow S_j$. Similarly, if $r^* \notin S$, set $j \leftarrow \text{search}(S, r^*)$ and $S_{r^*} \leftarrow S_j$. Then, if $S_l \neq 2$ go to C6.

C5. [Segment MRCA] Set $S_\ell \leftarrow 0$ and $r \leftarrow \text{nextkey}(S, \ell)$. Go to C7.

C6. [Decrement overlaps.] Set $r \leftarrow \ell$. Then, while $S_r \neq 2$ and $r < r^*$, set $S_r \leftarrow S_r - 1$ and $r \leftarrow \text{nextkey}(S, r)$. Afterwards, set $\alpha \leftarrow \text{Segment}(\ell, r, u)$.

C7. [Update x and y] Set $C \leftarrow C \cup \{(\ell, r, \text{node}(x), \text{node}(y), u, t)\}$. If $\text{right}(x) = r$, set $x \leftarrow \text{next}(x)$; otherwise, set $\text{left}(x) \leftarrow r$. If $\text{right}(y) = r$, set $y \leftarrow \text{next}(y)$; otherwise, set $\text{left}(y) \leftarrow r$.

C8. [Update links] If $\alpha = \Lambda$ go to C2. If $z = \Lambda$ set $P \leftarrow P \cup \{\alpha\}$ and $L_\alpha \leftarrow \text{right}(\alpha) - \text{left}(\alpha) - 1$; otherwise, set $\text{next}(z) \leftarrow \alpha$ and $L_\alpha \leftarrow \text{right}(\alpha) - \text{right}(z)$. Afterwards, set $\text{prev}(\alpha) \leftarrow z$, $z \leftarrow \alpha$ and go to C2

We begin in step C1 by choosing our individuals x and y and removing them from the set of ancestors. We then set the tail of the segment chain representing the common ancestor z to the null segment Λ, and then proceed into the main loop of the algorithm. This loop is controlled in step C2, and works by taking the leading segment from the x and y chains at each iteration and processing it. Once all segments have been consumed, we exit. Therefore, if both x and y are null, this loop has completed and we terminate the algorithm. Otherwise, we set α to the null segment. Throughout, we use this variable to point to the next segment that is to be merged into the segment chain representing the ancestor of the two chosen individuals. The last-merged segment in this chain is pointed to by z, and the necessary operations to include α into the global state are carried out in step C8.

Returning to the head of the loop in C2, if either x or y is null we have reached the end of one of the segment chains, and all that remains to do is attach the remainder of the non-null chain to our new individual. If both x and y are non-null, on the other hand, we proceed to C3. In this step we consider the two segments x and y and decide which of a number of cases we must deal with. First, we maintain the invariant that $\text{left}(x) \leq \text{left}(y)$; if this is violated, we swap the variables. Then, we address the various cases that can occur as x and y overlap.

The simplest case is when there is no overlap between x and y which occurs when $\text{right}(x) \leq \text{left}(y)$; here, we simply merge x into the new segment chain and move on to C8. The next case we deal with is when we have a partial overlap between x and y, which occurs when $\text{left}(x) \neq \text{left}(y)$. In this case, we create a new segment to represent this 'overhang', and merge this into the new segment chain in C8. Finally, if none of these conditions have been satisfied, we know that $\text{left}(x) = \text{left}(y)$ and there is therefore a coalescence which we handle in C4.

First, we check if another coalescence has occurred during this common ancestor event. If not, we set our flag $c \leftarrow 1$, and increment the next node w. Afterwards, we set the parent node for this coalescence u, and set ℓ and r^* to

the boundaries of the coalescing interval. We then check if ℓ and r^* are in S so that we can subsequently update the number of extant segments in the intervals to account for the coalescence. There are then two possibilities: if $S_\ell = 2$, we know that the MRCA has been reached in an interval starting at ℓ, which we deal with in C5; if not, we move on to C6.

In general, there will be many intervals with different numbers of extant segments overlapping between ℓ and r^*. In C6 we iterate over each of these intervals, decrementing the number of extant segments to account for the current coalescence. After this has completed, we allocate the new segment α and move on to C7. Here, we record the coalescence by updating the set C, handle any trailing overlaps that may occur, and update x and y to point to the appropriate next segments in their respective chains.

Step C8 is the final step of the main loop, where we insert the new segment α into the chain representing the common ancestor. Firstly, if this segment is null as a result of reaching the MRCA, then we have nothing to do, and so return to the start of the main loop. The variable z is used to keep track of the previous segment that was merged into the common ancestor's segment chain. Thus, if z is null we know that α is the first segment in the new chain and so we can use this opportunity to insert the new individual into the set of ancestors P; otherwise, we merge α into the existing chain. In both cases, we update the number of links subtended by α as appropriate, before returning to C2.

As stated, Algorithm H correctly simulates the coalescent with recombination and returns a set of coalescence records fully describing the generated genealogies. In the interest of brevity we have omitted some details that are important for efficiency. Firstly, it is important to defragment segments in order to save time and memory. That is, if we have two adjacent segments (ℓ, k, u) and (k, r, u) we should merge these into a single equivalent segment (ℓ, r, u). This can be done quite simply after Algorithm C has completed, and we can detect when such defragmentation is required in step C8. Similarly, it is vital for efficiency to opportunistically defragment the map that counts the number of extant segments in a given interval. Since S_j counts the number of segments covering the interval $[j, k)$, where k is the smallest key $> j$ in S, if $S_j = S_k$ we can simply delete the key k without loss of information. Although it does not affect simulation efficiency, it is also important to defragment the coalescence records output by the algorithm. This is easily done, since any records (ℓ, k, u, c, t) and (k, r, u, c, t) that can be merged must be stored sequentially without any intervening records.

Variable recombination rates can be incorporated into Algorithm H in (at least) two different ways. The most direct way (and the approach taken in `cosi2`) is to replace the uniform weight associated with segments when they are chosen as recombination breakpoints with a probability distribution. When we insert a segment z into the binary indexed tree L, we currently assume that all the links it subtends are equally likely to be the focus of a recombination event (see steps H1, R3, R4 and C8). To implement a variable recombination rate then, we need to assign the weights induced by the recombination map on the subtended links to a segment when we insert it into L. We also need

to modify step R1 to generate the location of a breakpoint following the correct distribution. The changes required to the algorithm to implement this are straightforward.

The second way to implement a variable recombination rate (and the approach currently taken in `msprime`) is to do so when we are generating mutations, which therefore does not require any modifications to Algorithm H. In this case we assume that the coordinates of coalescence records are in genetic space, which we can be transformed into physical coordinates using a given genetic map. Mutations are then generated in this physical coordinate space and mapped onto the appropriate genealogies. The advantage of this approach over the method outlined in the previous paragraph is that it is quite efficient to implement, since we only need to use the recombination map to translate back and forth between coordinate spaces during mutation generation. The disadvantage of this 'post-processing' approach is that other processes such as gene conversion are more difficult to incorporate.

Incorporating selection into coalescent simulations is non-trivial, but existing methods (Spencer and Coop, 2004; Teshima and Innan, 2009; Ewing and Hermisson, 2010; Shlyakhter et al., 2014) may be adapted to include specific forms of selection in Algorithm H.

The implementation of `msprime` is closely based on Algorithm H as given here. We also provide a simpler Python implementation in the file `algorithms.py` at `https://github.com/jeromekelleher/msprime-paper`. This repository also contains all code required to run the simulations, and to create all figures and illustrations in this paper.

References

G. Ewing and J. Hermisson. MSMS: a coalescent simulation program including recombination, demographic structure, and selection at a single locus. *Bioinformatics*, 26(16):2064–2065, 2010.

P. M. Fenwick. A new data structure for cumulative frequency tables. *Software: Practice and Experience*, 24:327–336, 1994.

P. M. Fenwick. A new data structure for cumulative frequency tables: an improved frequency-to-symbol algorithm. Technical Report 110, The University of Auckland, Department of Computer Science, 1995.

D. E. Knuth. *Sorting and Searching*, volume 3 of *The Art of Computer Programming*. Addison-Wesley, Reading, Massachusetts, second edition, 1998.

I. Shlyakhter, P. C. Sabeti, and S. F. Schaffner. Cosi2: an efficient simulator of exact and approximate coalescent with selection. *Bioinformatics*, 30(23): 3427–3429, 2014.

C. C. Spencer and G. Coop. SelSim: a program to simulate population genetic

data with natural selection and recombination. *Bioinformatics*, 20(18):3673–3675, 2004.

K. M. Teshima and H. Innan. mbs: modifying hudson's ms software to generate samples of DNA sequences with a biallelic site under selection. *BMC Bioinformatics*, 10(1):166, 2009.
