# Supplementary material for: Efficient Coalescent Simulation and Genealogical Analysis for Large Sample Sizes
Source: PLoS Comput Biol. 2016 May 4;12(5):e1004842. doi: 10.1371/journal.pcbi.1004842 (PMC4856371; doi:10.1371/journal.pcbi.1004842)
Supplement: S2 Text — (PDF) [file pcbi.1004842.s002.pdf]

Illustration of Hudson's algorithm

Fig. 1 shows an illustration of Hudson's algorithm for a sample of four individuals. In this illustration we show the state of the algorithm and its effects on the marginal trees after every event. The state of the algorithm is fully defined by the ancestral lineages (defined by the segments of ancestral material that they carry), the next available node w and the current time t. Although it is not necessary to store the partially built genealogies in memory, we show them here in the lower part of each panel for clarity. The left-to-right axis represents genomic coordinates. We also show the current time (t) and the number of potential recombination breakpoints (B) in each panel.

In this example, we have simulated the ancestry of the sample for a sequence of 10 sites. The initial state of the simulation at time 0 is shown in panel (a), where we see four lineages corresponding to our sampled chromosomes. Lineage l_1 can be represented as the segment $(0, 10, 1)$, which states that over the genomic interval $[0, 10)$, the lineage occupies the tree node 1. This information is shown explicitly in the figure, where we draw the full range of each segment and label the line with the node with which it is associated. Nodes are colour-coded, so that we can easily see which tree nodes are associated with each segment. Since this is the initial state of the algorithm, the only tree nodes defined are the leaf nodes. This is shown in the bottom part of the panel, where we draw out the nodes of the trees that have been assigned so far. (The nodes are ordered in these panels such that they are consistent with the orderings induced by later events.)

The first event that we encounter as we go backwards in time is a recombination event which occurs at time $t = 0.007$. Panel (b) shows the state of the simulation immediately after this event. Recombination has split lineage l_3, resulting in two new lineages, l_5 and l_6. As the breakpoint was at 2, we have $l_5 = (0, 2, 3)$ and $l_6 = (2, 10, 3)$. The other effect of this recombination event is to create a new tree: since the histories of the sample over the intervals $[0, 2)$ and $[2, 10)$ can now be different, we must create a new tree to record these histories as they are simulated. (Note again that these trees are shown for illustration only; they are not stored in the simulation.)

After the recombination in event (b), a common ancestor event occurs in (c) in which l_4 and l_6 are merged to form a common ancestor l_7. At a common ancestor event we merge the ancestral material from two lineages. Any non-overlapping segments are copied directly into the new lineage. In this example, only one of the lineages carried ancestral material in the interval $[0, 2)$, and so this is copied directly to the common ancestor. However, in the interval $[2, 10)$ both carry ancestral material, and so a coalescence occurs. In this coalescence, nodes 3 and 4 have a common ancestor in the interval $[2, 10)$. We therefore create a new node 5, and update the tree covering the interval $[2, 10)$ to reflect this.

The simulation continues generating common ancestor and recombination events at the relevant rates until complete genealogies have been generated across the entire sequence. Termination of the algorithm is controlled by keep-

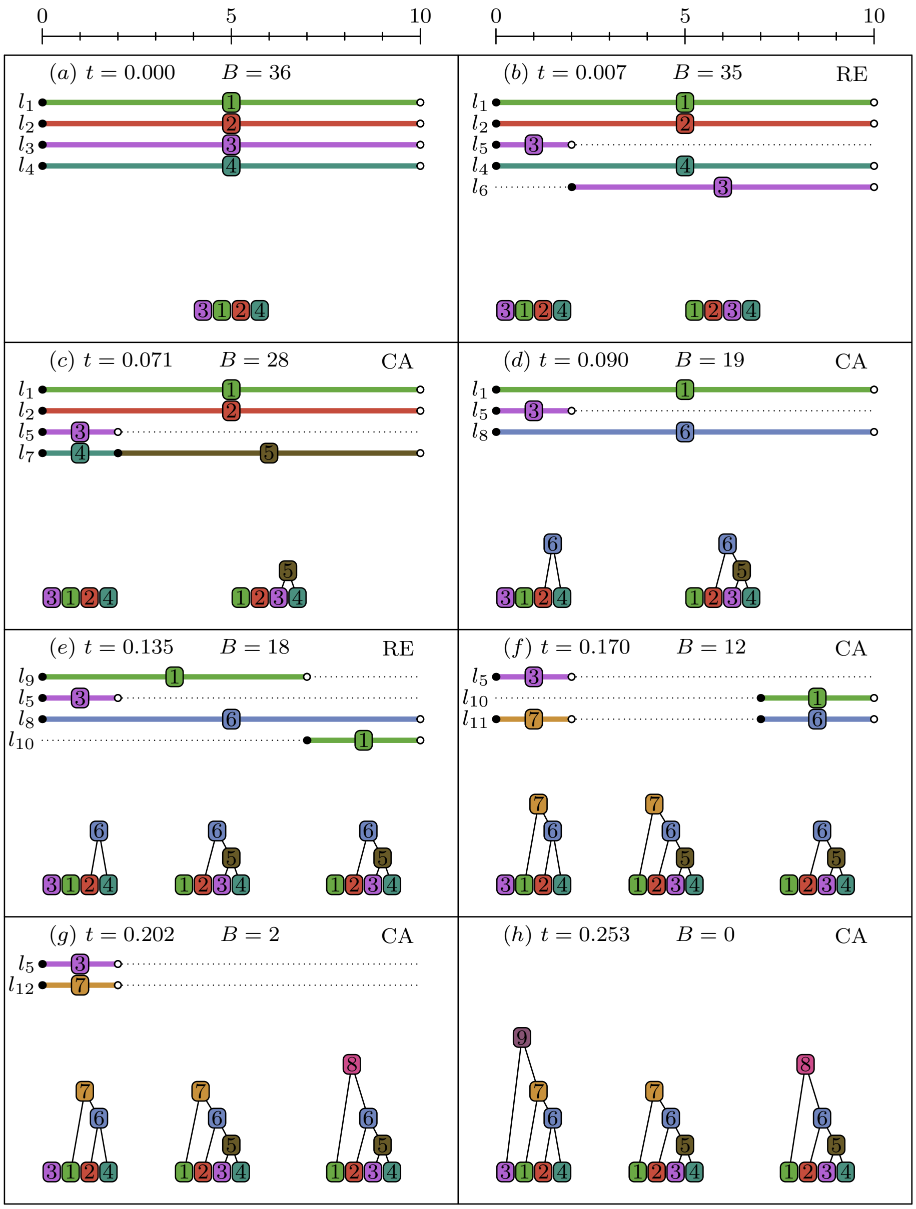

Figure 1: An illustration of Hudson's algorithm using sparse trees. In each panel we show the state of the algorithm after an event. Events are either recombination (RE) or common ancestor (CA). On the top of each panel, every line represents an ancestor which may be composed of several distinct segments. The bottom of each panel shows the state of the trees at that point in time. The horizontal direction represents genomic coordinates.

	**l**	**r**	**u**	**c**	**t**
1	2	10	5	(3, 4)	0.071
2	0	2	6	(2, 4)	0.090
3	2	10	6	(2, 5)	0.090
4	0	7	7	(1, 6)	0.170
5	7	10	8	(1, 6)	0.202
6	0	2	9	(3, 7)	0.253

Table 1: Tabular representation of the coalescence records output by the simulation in Fig. 1. The corresponding index vectors are $\mathcal{I} = (2, 4, 6, 1, 3, 5)$ and $\mathcal{O} = (6, 2, 4, 5, 3, 1)$.

ing track of the amount of ancestral material present in each distinct interval produced by recombination. An important aspect of Hudson's algorithm is that we do not continue to track the ancestry of segments in which the trees are already complete. An example of this can be seen in panel (f) of Fig. 1. In this event lineages l_8 and l_9 have merged to form l_{11}. In the interval $[0, 7)$, these have overlapping ancestral material and we therefore create a new node 7 and update the trees covering $[0, 2)$ and $[2, 7)$ to show that node 7 is the parent of 1 and 6. However, we note that the tree covering the interval $[2, 7)$ is complete as a result, and so we omit the segment mapping to the new node over this interval. This process is important for efficiency, as we would continue to generate recombination and common ancestor events for the segment, even though these events could not effect the genealogy over this interval.

Panel (f) also illustrates the concept of trapped material. Lineage l_{11} consists of the two segments $(0, 2, 7)$ and $(7, 10, 6)$. Recombination events occurring anywhere in $[0, 10)$ on this lineage will therefore result in a different arrangement of ancestral material. The total number of possible recombination breakpoints for l_{11} is therefore 9. In contrast, there are only 2 possible breakpoints for l_{10}, since any recombination that occurs in $[0, 7)$ cannot affect the ancestral material. Similarly l_5 has only one potential breakpoint, and so the total number of potential breakpoints $B = 12$.
