# Supplementary figures and images for: Efficient Coalescent Simulation and Genealogical Analysis for Large Sample Sizes
Source: PLoS Comput Biol. 2016 May 4;12(5):e1004842. doi: 10.1371/journal.pcbi.1004842 (PMC4856371; doi:10.1371/journal.pcbi.1004842)

sample size = 1000

 $\rho \times 10^3$ 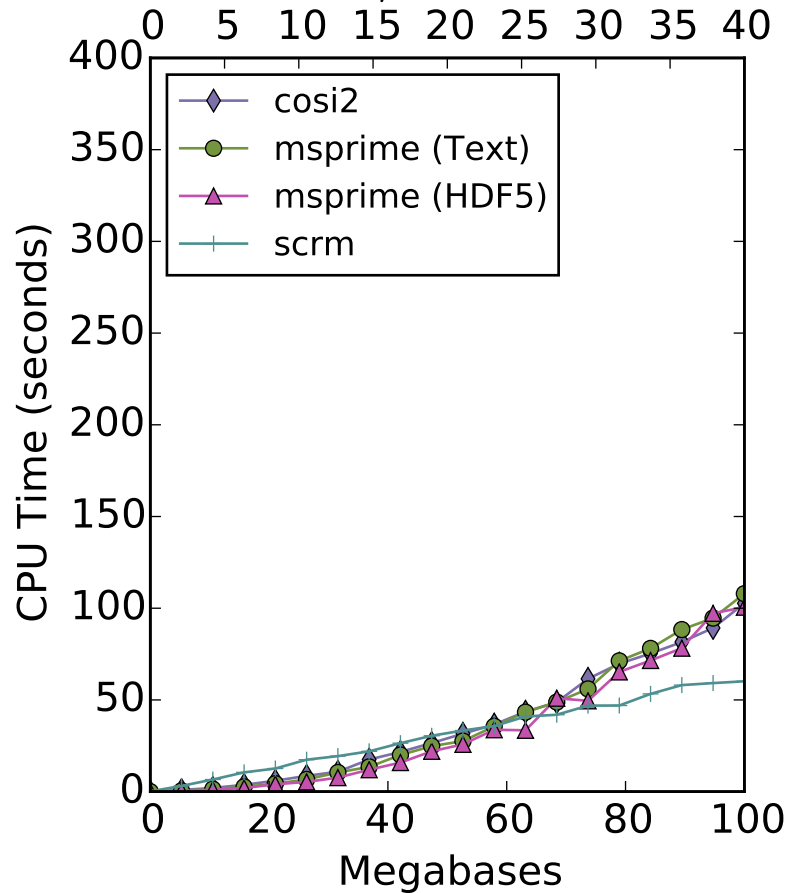

sequence length = 50Mb

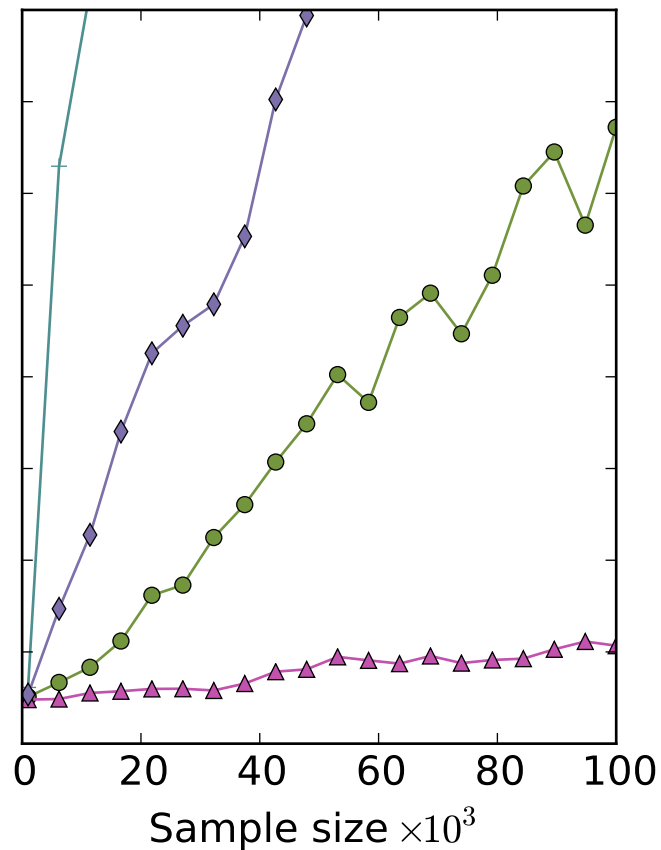

Supplement: S1 Fig — We use a scaled mutation rate of θ = 4Neμ = 0.0004. (PDF) [file pcbi.1004842.s003.pdf]

sample size = 1000

 $\rho \times 10^3$ 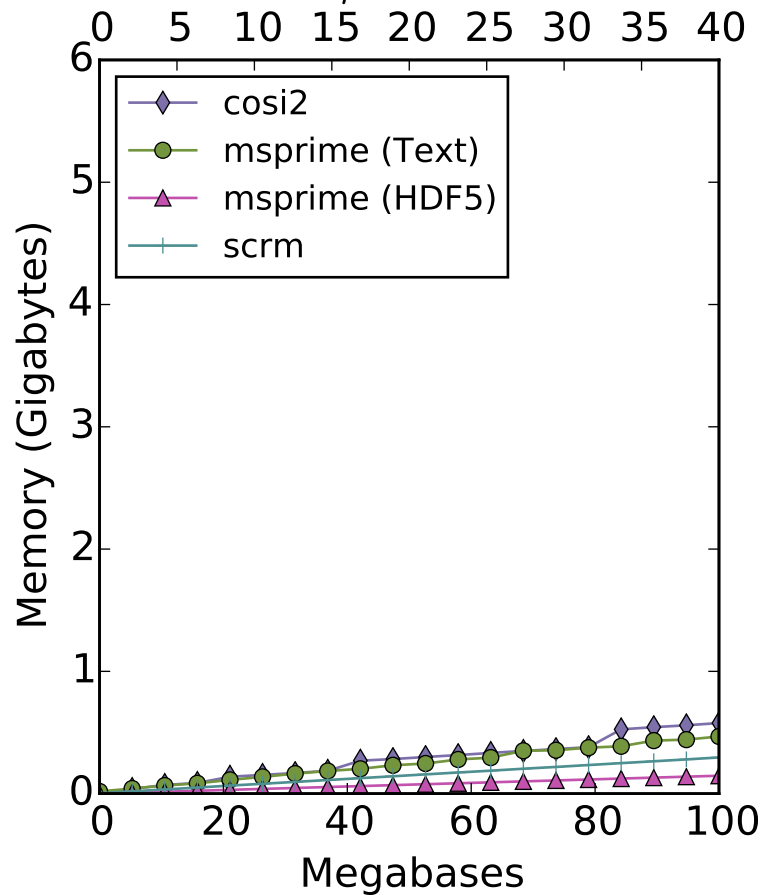

sequence length = 50Mb

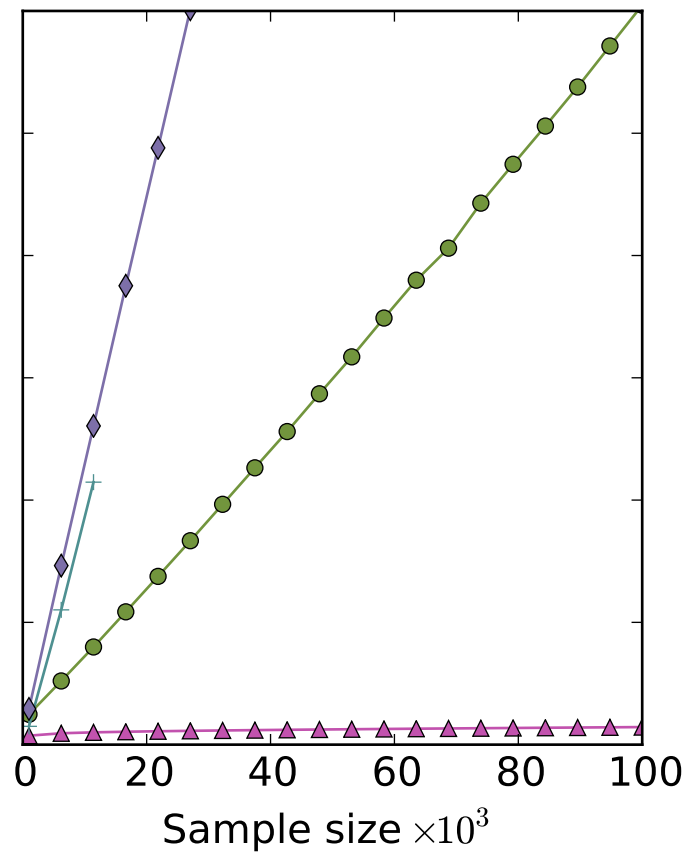

Supplement: S2 Fig — (PDF) [file pcbi.1004842.s004.pdf]

sample size = 1000

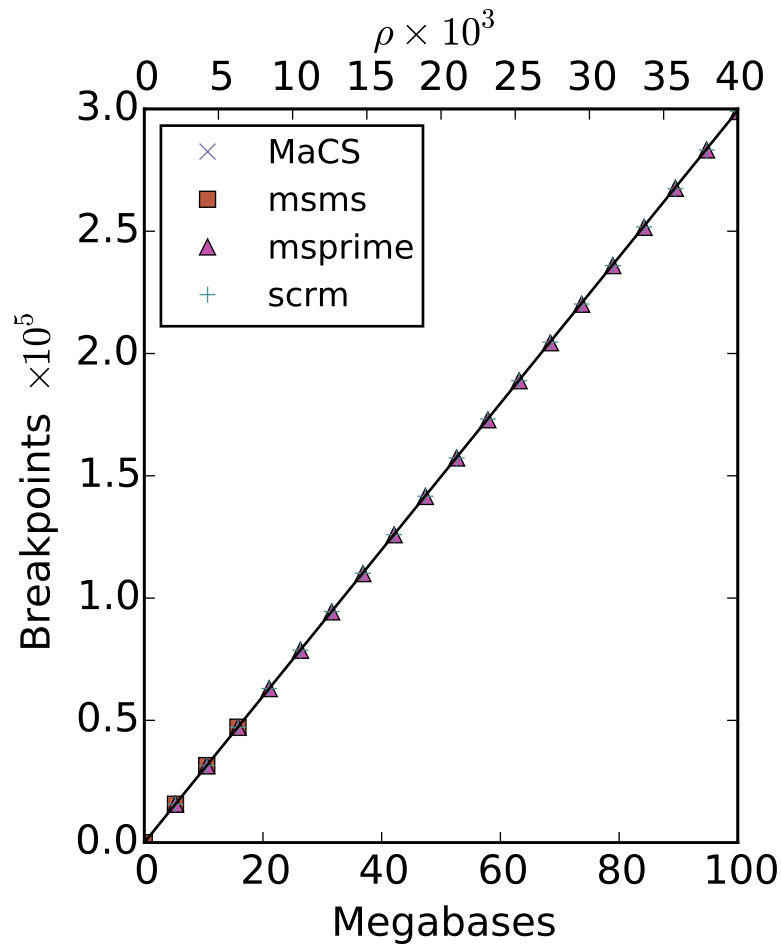

sequence length = 50Mb

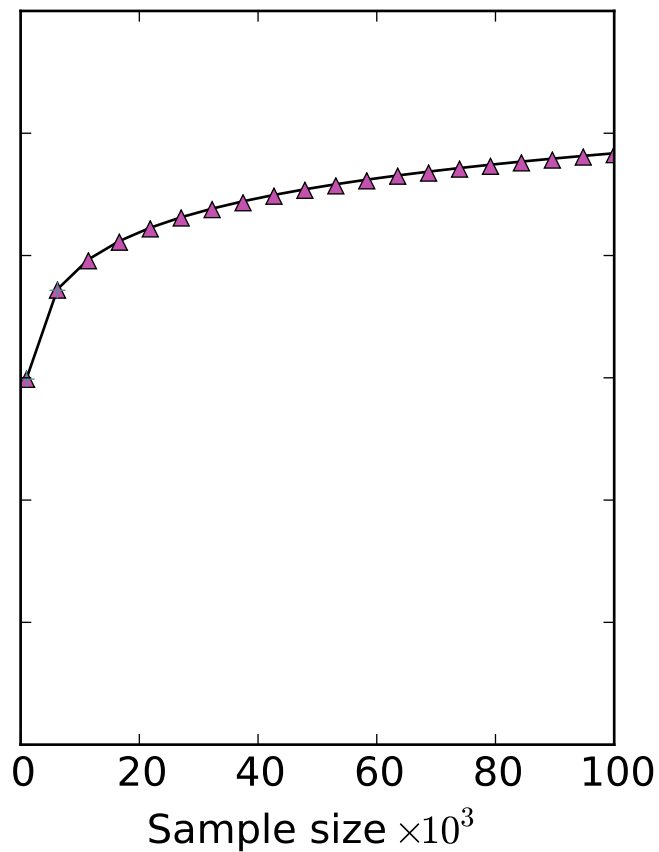

Supplement: S3 Fig — This plot shows that the number of recombination events within ancestral material for these simulations is identical for all simulators and agrees very well with the theoretical value of ρHn − 1, where Hn is the nth Harmonic number. (PDF) [file pcbi.1004842.s005.pdf]

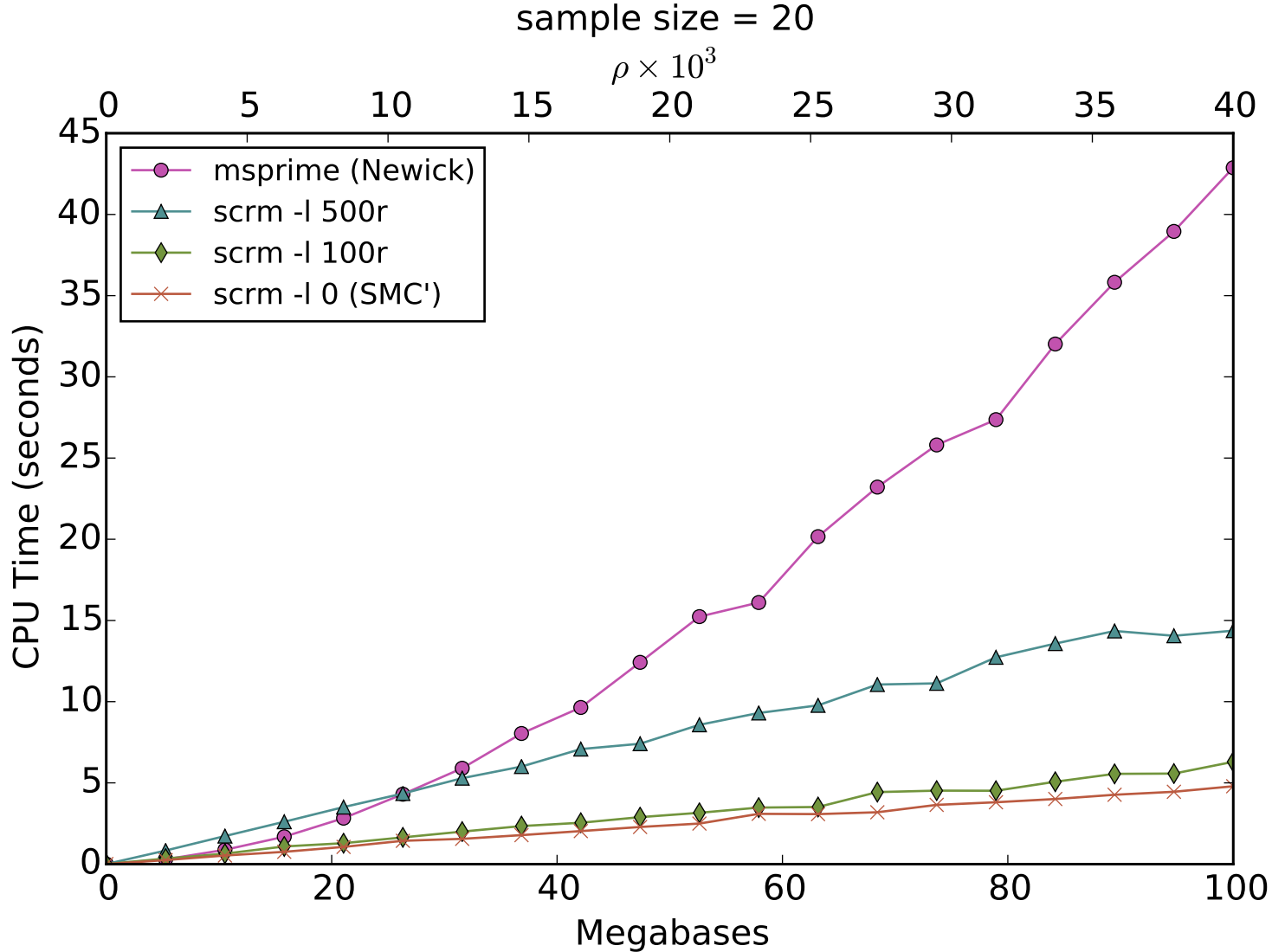

Supplement: S4 Fig — Several different approximation levels are shown for scrm using the -l option. The -l 500r option is described as a conservative value giving very good accuracy, and -l 100r is recommended as a good compromise between running time and accuracy. (PDF) [file pcbi.1004842.s006.pdf]
